# Comparison of the Effectiveness of Ondansetron and Domperidone in Cessation of Vomiting in Children Presenting With Acute Gastroenteritis: A Meta-Analysis

**DOI:** 10.7759/cureus.27636

**Published:** 2022-08-03

**Authors:** FNU Aisha, Kanwal Bhagwani, Huda Ijaz, Krupali Kandachia, Naresh Kumar, Sana Faisal, Saswat Jha, Samiullah Khan

**Affiliations:** 1 Medicine, Medical College, Liaquat University of Medical and Health Sciences, Hyderabad, PAK; 2 Medicine, Chandka Medical College, Larkana, PAK; 3 Internal Medicine, Allama Iqbal Medical College, Lahore, PAK; 4 Medicine, Gujarat Medical Education and Research Society (GMERS) Medical College, Vadodara, IND; 5 Medicine, Liaquat National Hospital and Medical College, Karachi, PAK; 6 Family Medicine, Medical College, Dow University of Health Sciences, Karachi, PAK; 7 Internal Medicine, Kathmandu Medical College and Teaching Hospital, Kathmandu, NPL; 8 Emergency Medicine, Medical College, Baqai University, Karachi, PAK

**Keywords:** pediatrics, vomiting, ondansetron, domperidone, acute gastroenteritis

## Abstract

Acute gastroenteritis is one of the common diseases of childhood. Dehydration is the most frequent consequence of acute gastroenteritis, and vomiting is the most distressing clinical manifestation. Various anti-emetic agents are used in practice to control vomiting. However, not all anti-emetic agents are safe and effective. This meta-analysis aims to compare the effectiveness of ondansetron and domperidone in the cessation of vomiting in children with acute gastroenteritis. The current meta-analysis was conducted using the Preferred Reporting Items for Systematic Reviews and Meta-Analyses (PRISMA) guidelines. A comprehensive search strategy was developed to identify prospective studies that compared the effectiveness of ondansetron and domperidone in the cessation of vomiting in children with acute gastroenteritis. The primary outcome was the number of children in whom there was a cessation of vomiting. The secondary outcomes included a number of children who required an additional dose of the assigned anti-emetic and the number of children who required intravenous rehydration therapy. Overall, seven randomized trials were included in the current meta-analysis. The pooled sample size of enrolled patients was 1,262, of which 639 patients were randomized to the ondansetron group and 623 were randomized to the domperidone group. In the ondansetron group, a higher number of children experienced cessation of vomiting (risk ratio [RR]: 1.22, 95% CI: 1.08-1.37, p-value=0.002), a lower proportion of children needed an additional dose of the assigned anti-emetic (RR=0.50, 95% CI: 0.33-0.77, p-value=0.002), and a lower number of children received intravenous rehydration (RR: 0.37, 95% CI: 0.16-0.83, p-value=0.02) as compared to domperidone group. Compared to domperidone, ondansetron was found to have better efficiency in aiming cessation of vomiting in children with gastroenteritis.

## Introduction and background

Acute gastroenteritis is one of the common diseases of childhood, which accounts for 13% of hospitalization for children younger than five years [1]. Even though the mortality rate because of gastroenteritis in developed countries is low, dehydration and diarrhea are common causes of mortality. The number of children under the age of five who died from diarrhea over the world was estimated at 1.87 million [2]. Vomiting is the most aggravating clinical symptom of acute gastroenteritis, and dehydration is its most common consequence. Vomiting can lead to dehydration, which may require emergency medical care, and it is a source of distress and worry for both the child and the caregiver [3]. To treat vomiting and diarrhea in children with acute gastroenteritis, a variety of interventions have been suggested [4]. Currently, no guidelines are there for the utilization of pharmacological treatment in the management of vomiting for children with acute gastroenteritis [5]. The World Health Organization (WHO) recommended oral rehydration solution (ORS) as the treatment of choice for children with mild-to-moderate gastroenteritis [6]. However, this mode of treatment is limited by the accompanying vomiting. Therefore, when a child has gastroenteritis, it is crucial for healthcare professionals to properly treat the vomiting because it helps children to drink fluids orally, potentially reducing the need for intravenous therapy [7].

Various anti-emetic agents are used in practice to control vomiting. However, not all anti-emetic agents are safe and effective [8]. Domperidone is frequently used to prevent vomiting in young children; however, the efficiency of this treatment has not yet been conclusively proven [9]. Numerous studies provide more support for the use of ondansetron to reduce vomiting in acute gastroenteritis [7,9-10].

There has not been much research comparing the effectiveness of ondansetron with domperidone utilizing multicenter large sample randomized controlled trials (RCTs). Additionally, other investigations produced contradictory findings [9-10]. Thus, the objective of the current meta-analysis was to provide a comprehensive response to the topic utilizing a large sample size. The main aim of this meta-analysis is to compare the effectiveness of ondansetron and domperidone in the cessation of vomiting in children with acute gastroenteritis.

## Review

Methodology

Preferred Reporting Items for Systematic Reviews and Meta-Analyses (PRISMA) guidelines were used to conduct this meta-analysis. A comprehensive search strategy was developed to identify prospective studies that compared the effectiveness of ondansetron and domperidone in the cessation of vomiting in children with acute gastroenteritis. A search was conducted using different online databases including PubMed, Cochrane Library, and Embase. Key terms used included “ondansetron,” “domperidone,” “acute gastroenteritis,” “vomiting,” and “clinical outcomes” either separately or in combination. No restrictions were placed on the year of publication. Two investigators reviewed all the titles and abstracts from the primary search and assessed the articles for eligibility criteria. Any discrepancy between two investigators is resolved via consensus or involvement of a third investigator.

Eligibility Criteria

We included prospective studies that compared the effectiveness of ondansetron and domperidone in reducing vomiting in children with acute gastroenteritis. We excluded all studies that compared ondansetron or domperidone with placebo or any other medication. Review articles, editorials, meta-analyses, observational studies, and published abstracts were excluded from the present meta-analyses. Studies that were published in a language other than English were also excluded from the current meta-analysis.

Assessment of Risk of Bias

All eligible studies were assessed for methodological validity by two independent reviewers. For this purpose, Cochrane Handbook version 5.0.2 was used. Information assessed for this purpose included "blinding, random sequence generation, selective reporting, allocation concealment, and other kinds of biases." The risk of bias graph describes all judgments and was drawn using Review Manager (Revman) software version 5.4.1 (The Cochrane Collaboration, Copenhagen, Denmark). Any kind of discrepancy between the two reviewers was resolved via consensus or involvement of a third reviewer.

Outcomes

The primary outcome was the number of children in whom there was a cessation of vomiting. The secondary outcomes included the number of children who required an additional dose of the assigned anti-emetic and a number of children requiring intravenous rehydration therapy.

Data Extraction

Data were extracted independently by two authors for each article using a standardized table. Data extracted from articles included author name, year of publication, sample size, medication dose, inclusion criteria, study outcomes, follow-up time, and participant characteristics (age, weight, gender, and height).

Statistical Analysis

Mantel Haenszel's random-effects model was used to calculate the risk ratio (RR) and corresponding 95% confidence interval for primary and secondary endpoints. Publication and small study bias were assessed with Egger’s regression test. P-values less than 0.05 were considered significant. *I*^2^ statistics were calculated to evaluate heterogeneity. Cochran's Q test was used for statistical testing of heterogeneity. A p-value less than 0.1 will be considered significant for heterogeneity. Statistical analyses were performed using Stata (Version 16, StataCorp, College Station, Texas) and RevMan software version 5.4.1 (The Cochrane Collaboration, Copenhagen, Denmark).

Results

**Figure 1:**
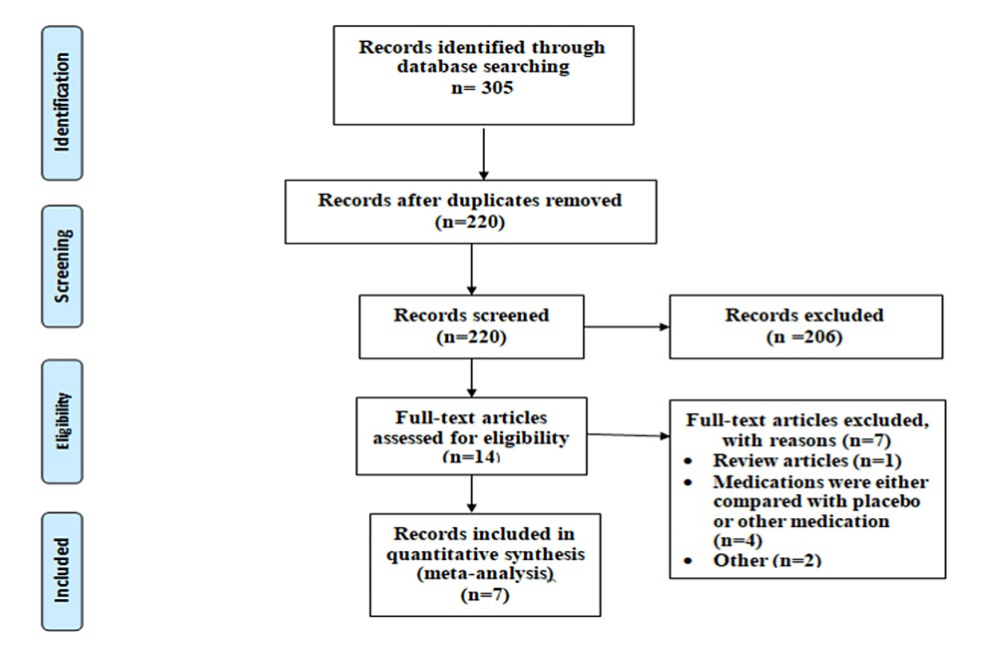
Flow chart for article selection in the meta-analysis.

Figure 1 shows the PRISMA chart of the selection of studies. The search strategy identified 305 studies. After removing duplicates, 220 studies were retained for a title and abstract screening. In total, 14 studies were selected for full-text screening, out of which 7 studies were retained in the current meta-analysis [7,9-14].

The characteristics of included studies are shown in Table 1. Studies were performed in the emergency department and had follow-up times ranging from 24 hours to 7 days during which patients were asked to report symptoms and further management use. Route of administration was oral in all of the included studies. Regarding the dosage of medications, four studies used a dose of 0.15 mg/kg and 0.5 mg/kg of body weight of the ondansetron and domperidone group, respectively [10-12,14]. In one trial, fixed weight-based dosing was used [9]. The dosing of the two studies was not mentioned in the articles [7,13].

**Table 1:** Characteristics of included studies AGE, acute gastroenteritis

Author	Year	Groups	Sample Size	Dose	Follow-up	Setting	Route of administration	Inclusion criteria
Hanif et al. [11]	2019	Ondansetron	123	0.15 mg/kg of body weight	24 Hours	Emergency	Orally	All children aged five years or less and who experienced at least three vomiting episodes in a 24-hour period along with symptoms of acute gastroenteritis such diarrhea, stomach pain, bloating, or discomfort—fever included—were included.
		Domperidone	117	0.5 mg/kg of body weight				
Iqbal et al. [7]	2022	Ondansetron	100	-	24 Hours	Emergency	Orally	Children between 6 months to 5 years of age having acute diarrhea with or without abdominal pain and fever, with three or more episodes of vomiting not containing blood or bile, in 24 hours, were included in the study
		Domperidone	98	-				
Ibrahim et al. [12]	2022	Ondansetron	75	0.15 mg/Kg of body weight	24 Hours	Emergency	Orally	This study included children who experienced diarrhea in the previous 24-48 hours
		Domperidone	75	0.5 mg/Kg of body weight				
Kamal et al. [13]	2015	Ondansetron	42	-	7 days	Pediatric ward	Oral	Children having AGE and aged 3-12 years
		Domperidone	42	-				
Marchetti et al. [10]	2016	Ondansetron	119	0.15 mg/kg of body weight	48 Hours	Emergency	Oral	Children aged 1 to 6 years who had vomiting, with a presumptive clinical diagnosis of AG, and without severe dehydration
		Domperidone	119	0.5 mg/kg of body weight				
Rerksuppaphol and Rerksuppaphol [9]	2013	Ondansetron	38	2 mg for children weighing less than 15 kg, 4 mg for children weighing 15 - 30 kg, and 8 mg for children weighing more than 30 kg.	72 hours	Pediatric clinics	Oral	Children aged 15 years or less presenting with symptoms of acute gastroenteritis
		Domperidone	38	The prescribed dose was 2.5 mg for children weighing less than 15 kg, 5 mg for children weighing 15-30 kg, and 10 mg for children weighing more than 30 kg.				
Ahmad et al. [14]	2022	Ondansetron	142	0.15 mg/kg of body weight	24 Hours	Emergency	Oral	All children under the age of 12 years who experienced at least three vomiting episodes in a 24-hour period along with symptoms of acute gastroenteritis
		Domperidone	134	0.5 mg/kg of body weight				

**Figure 2:**
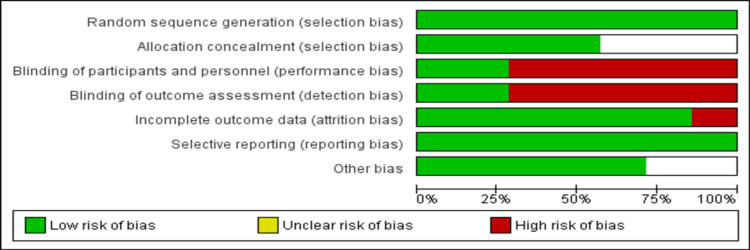
Risk of bias graph

The pooled sample size of enrolled patients was 1,262, of which 639 patients were randomized to the ondansetron group and 623 were randomized to the domperidone group. Two studies were designated as “low risk” for overall bias, three studies were designated as “high risk” for overall bias, and two studies were marked as being of “moderate risk” in the Cochrane Collaboration risk-of-bias assessment. The risk of bias assessment graph is shown in Figure 2.

Characteristics of Participants

Baseline patient characteristics for each study are summarized in Table 2. The mean age of participants ranged from 28 to 58.8 months. In all trials, more than 40% of enrolled participants were males.

**Table 2:** Characteristics of participants *Mean (standard deviation)

Study	Groups	Participants characteristics				
		Age (in months)*	Number of males	Body weight (kg)*	Height (cm)*	Mild-to-moderate dehydration
Hanif et al., 2019 [11]	Ondansetron	28 (15)	66 (53.7%)	13.4 (5.7)	96.5 (21.4)	85 (69.1%)
	Domperidone	27 (19)	53 (45.3%)	13.9 (6.2)	95.3 (20.5)	72 (61.5%)
Ibrahim et al., 2022 [12]	Ondansetron	51.6 (26.04)	30 (40%)	15.07 (8.23)	98.07 (11.14)	-
	Domperidone	36.96 (82.56)	33 (44%)	15.02 (3.09)	97.67 (18.24)	-
Iqbal et al., 2022 [7]	Ondansetron	-	-	-	-	-
	Domperidone	-	-	-	-	-
Kamal et al., 2015 [13]	Ondansetron	-	-	-	-	-
	Domperidone	-	-	-	-	-
Marchetti et al., 2016 [10]	Ondansetron	37.2 (15.6)	57 (47.9%)	14.2 (6.7)	98.5 (15.2)	56 (47.1%)
	Domperidone	38.4 (18.2)	65 (54.6%)	14.5 (5.8)	99.0 (22.2)	50 (42.0%)
Rerksuppaphol and Rerksuppaphol, 2013 [9]	Ondansetron	44.4 (6.0)	20 (52.6%)	16.7 (1.7)	99.1 (3.6)	
	Domperidone	56.4 (6.0)	21 (55.3%)	17.8 (1.3)	106.8 (2.9)	
Ahmad et al., 2022 [14]	Ondansetron	55.2 (26.4)	84 (56.0%)	-	-	102 (68.0%)
	Domperidone	58.8 (30)	78 (52.0%)	-	-	109 (72.7%)

Outcomes Comparison Between Ondansetron and Domperidone

**Figure 3:**
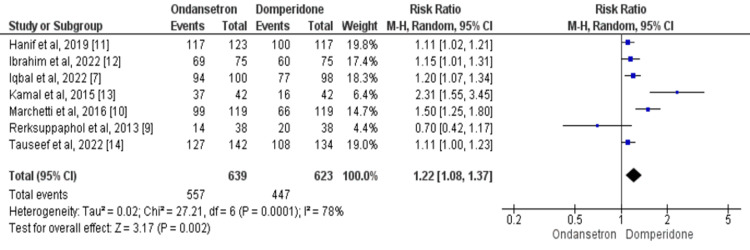
The risk ratio (RR) of cessation of vomiting in ondansetron-treated patients compared to domperidone. Each box's size varies according to the study's sample size. Diamond represents the pooled estimate, the peak of the diamond is the point estimate, and the horizontal lines of the diamond represent the 95% confidence intervals (CI). Source: [7,9-14]

Cessation of vomiting: Seven studies reported a number of children in whom there was a cessation of vomiting within 24 hours of administration of the study drug [7,9-14]. Using data from 1,262 patients, the cessation of vomiting is 1.22 times higher in the ondansetron group as compared to the domperidone group (95% CI: 1.08-1.37, p-value=0.002), as shown in Figure 3. There was a significant heterogeneity in the study results as *I*^2^=78% (p-value=0.001). The results of the Eggers test indicated no significant risk of publication bias for the outcome (p-value=0.826).

**Figure 4:**
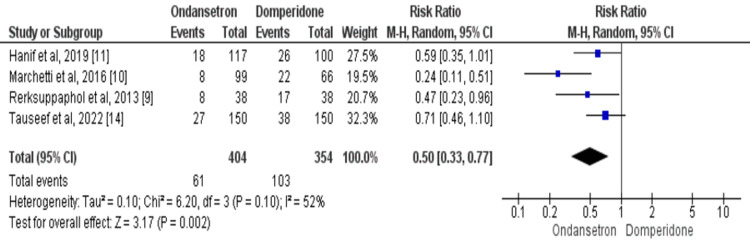
The risk ratio (RR) of need of additional dose of assigned anti-emetic in ondansetron-treated patients compared to domperidone. Each box's size varies according to the study's sample size. Diamond represents the pooled estimate, the peak of the diamond is the point estimate, and the horizontal lines of the diamond represent the 95% confidence intervals (CI). Sources: [9-11,14]

Subjects requiring additional dose of the assigned anti-emetic: Four studies reported a number of children required an additional dose of the assigned anti-emetic in the emergency department [9-11,14]. Using data from four studies, the proportion of children in the ondansetron group who need an additional dose of the assigned anti-emetic is lower as compared to the domperidone group (RR=0.50, 95% CI: 0.33-0.77, p-value=0.002), as shown in Figure 4. There was significant heterogeneity in the study results as *I*^2^=52% (p-value=0.001). The results of the Eggers test indicated no significant risk of publication bias for the outcome (p-value=0.724).

**Figure 5:**
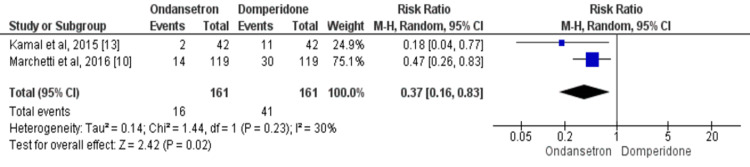
The risk ratio (RR) of need of intravenous rehydration in ondansetron-treated patients compared to domperidone. Each box's size varies according to the study's sample size. Diamond represents the pooled estimate, the peak of the diamond is the point estimate, and the horizontal lines of the diamond represent the 95% confidence intervals (CI). Sources: [10,13]

Subjects requiring intravenous rehydration: Two studies compared the number of children receiving rehydration therapy between two groups [10,13]. Using data from 322 patients, the number of children receiving intravenous rehydration is significantly lower in the ondansetron group as compared to the domperidone group (RR: 0.37, 95% CI: 0.16-0.83, p-value=0.02), as shown in Figure 5. There was insignificant heterogeneity in the study results as *I*^2^=30% (p-value=0.23). We did not assess publication bias in this outcome as the number of studies was less.

Sensitivity analysis: Sensitivity analysis was performed by including only those studies conducted in the emergency department [7,9-12,14]. The heterogeneity decreased from 78% to 54% after removing two studies carried out in the pediatrics inpatient ward and outpatient clinic. Results are similar to the overall analysis, as shown in Table 3.

**Table 3:** Results of sensitivity analysis RR, risk ratio; CI, confidence interval

Outcome	Included studies	RR (95% CI)	P-value	*I*^2^
Cessation of vomiting	[7,9-12,14]	1.18 (1.08-1.29)	0.001	64%
Required additional dose of the assigned anti-emetic	[10-11,14]	0.50 (0.28-0.88)	0.021	54%

Discussion

Gastroenteritis is one of the common childhood diseases, but comparatively few experimental studies related to drugs to treat vomiting have been conducted [15]. The current meta-analysis was conducted to compare the effectiveness of ondansetron with domperidone in reducing vomiting in children with acute gastroenteritis. The current meta-analysis showed that children who received ondansetron had less likely to have ongoing vomiting and fewer children in this group required an additional dose of the assigned anti-emetic and intravenous rehydration. In children with gastroenteritis who are unwell, the symptomatic improvement and avoidance of invasive interventions such as intravenous rehydration therapy or anti-emetic are significant outcomes that show a benefit of ondansetron treatment [15].

The current meta-analysis found that ondansetron has better efficiency as compared to domperidone for cessation of vomiting in children with gastroenteritis. It is possibly because of the fact that when ondansetron is taken orally, it is absorbed in the gastrointestinal tract and acts as a "serotonin 5-HT3 receptor antagonist," suppressing the brain's vomiting centers and blocking afferent depolarization of peripheral vagal nerves in the intestine that may be causing emesis responses in gastroenteritis patients [16-17]. As ondansetron is thought to lessen emesis, it may increase the oral uptake of fluids, thereby reducing the need for hospitalization and intravenous rehydration [18].

The utilization of several forms of ondansetron in children with vomiting and nausea has been subjected to different studies in the past. In all the seven studies included in the current meta-analysis, children were given ondansetron and domperidone via the oral route only. The oral disintegrating ondansetron offers a practical method of administration and is simpler than intravenous ondansetron to provide to children who are vomiting. Additionally, it is less invasive than intravenous ondansetron, particularly for kids who are supposed to receive outpatient care [15].

Ondansetron is an anti-emetic medication with consistent and proven efficiency in decreasing vomiting from gastroenteritis. Other anti-emetic medications should not be utilized because they have not shown consistent efficacy [15]. Besides this, moderately ill children who presented to the emergency children who were receiving ondansetron have a reduced hazard of receiving intravenous fluid and reduced need for hospital admission [19]. Without having any other negative effects, ondansetron probably increases the number of diarrheal bouts temporarily [15]. Government organizations and professional societies should seriously consider revising current gastroenteritis treatment recommendations to include the use of ondansetron for some children with gastroenteritis.

Even though this is the first comprehensive review of experimental study comparing the efficacy of ondansetron and domperidone in reducing vomiting in children with acute gastroenteritis, there are certain limitations to consider. Firstly, all studies have reported the effect of drugs on the cessation of vomiting, but other outcomes such as intravenous fluid requirement were not assessed by the majority of studies. Besides this, the current meta-analysis did not assess adverse events between the two groups due to the lack of experimental studies comparing safety profiles between ondansetron and domperidone. Future prospective and multicenter studies need to be conducted that compare safety outcomes between ondansetron and domperidone so that evidence can be gathered for implementation in the clinical setting.

## Conclusions

Compared to domperidone, ondansetron was found to have better efficiency in aiming cessation of vomiting in children with gastroenteritis. In addition, the percentage of children needing an additional dose of the assigned anti-emetic and intravenous fluid for rehydration purpose are also lower in the ondansetron group as compared to the domperidone group. It is important for public and private healthcare organizations and medical societies to seriously consider revising current gastroenteritis treatment recommendations to include the use of ondansetron for some children with gastroenteritis.
